# Persistent lingual ulceration (Riga-Fede disease) in an infant with Down syndrome and natal teeth: a case report

**DOI:** 10.1186/1752-1947-8-283

**Published:** 2014-08-22

**Authors:** Manouri P Senanayake, Irantha Karunaratne

**Affiliations:** 1 Department of Pediatrics, Faculty of Medicine, University of Colombo, Kynsey Road, Colombo 008, Sri Lanka; 2 University Unit, Lady Ridgeway Children’s Hospital, Gnarathapradeepa, Mawatte Borella, Colombo 008, Sri Lanka

**Keywords:** Riga-Fede disease, Down syndrome, Natal teeth

## Abstract

**Introduction:**

Riga-Fede disease is a rare pediatric condition in which chronic lingual ulceration results from repetitive trauma. Neonatal teeth or underlying neuro-developmental disorders which include Down syndrome are described as causative factors, but to the best of our knowledge, this is the first case report of both Down syndrome and natal teeth coexisting. The need for early extraction in the presence of two risk factors is highlighted in this case report.

**Case presentation:**

An 18-month-old Sinhalese male presented with an ulcerating lingual mass on the ventral surface of the tongue. The lesion had progressed over the past six months. He also had clinically diagnosed Down syndrome.

The ulcer was non-tender, indurated, and had elevated margins. It was not bleeding and two natal teeth in lower central dentition were seen in apposition with the lesion. There was no regional lymphadenopathy but the ulcer was causing concerns as it mimicked a malignant lesion. A clinical diagnosis of Riga-Fede disease caused by raking movements of the tongue against anterior natal teeth by a child who was developmentally delayed and prone to suck on his tongue was made. The mother was reassured and the natal teeth were extracted.

**Conclusions:**

Early extraction of natal teeth is recommended only if there is a risk of aspiration or interference with breast feeding. Although Down syndrome is among the neuro-developmental conditions that lead to this lesion, its occurrence is usually at an older age. The presence of natal teeth together with Down syndrome caused the lesion to occur in infancy. Awareness of the benign nature of this rare condition by pediatricians and dental practitioners is important as it will allay anxiety and avoid unnecessary biopsy. This case also highlights the impact of two risk factors and needs consideration as an added indication for the early extraction of natal teeth.

## Introduction

Riga-Fede disease is a rare entity seen in children and is characterized by persistent lingual ulceration due to repetitive mucosal trauma. First described in 1881 by the Italian physician Antonio Riga, its histology and benign nature were later described by Fede [1]. Neuro-developmental conditions are recognized to be associated with this condition. We describe a Sri Lankan male child with Down syndrome who had natal teeth and presented with a persistent lingual ulceration on the ventral surface of the tongue.

## Case presentation

**Figure 1:**
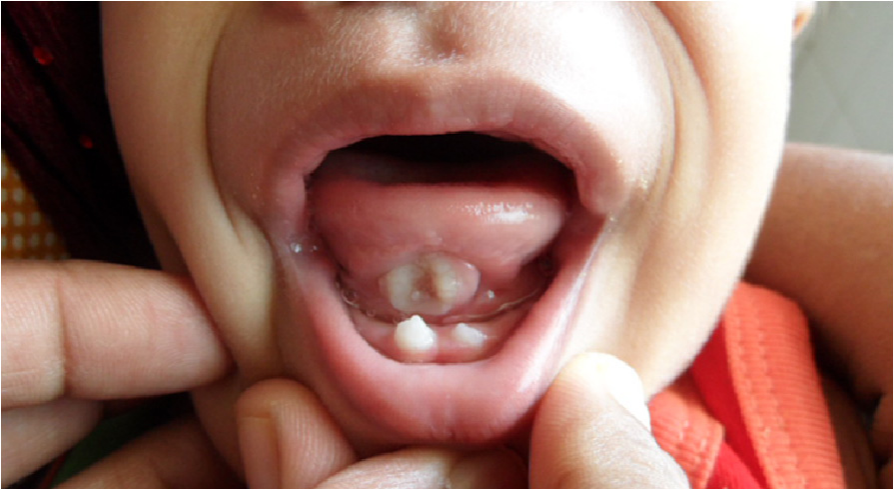
Ulcer on ventral surface of tongue and natal teeth (Riga-Fede disease).

**Figure 2:**
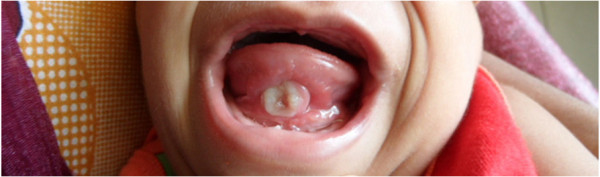
Riga-Fede disease in a child with Down syndrome.

An 18-month-old Sinhalese male with clinically diagnosed Down syndrome was presented to our institution due to concerns regarding a persistent ulcerating lingual mass on the ventral surface of his tongue. The ulcer had first been noticed around age of 11 months. He was otherwise in good health apart from occasional upper respiratory tract infections. There had been no difficulty in feeding and his tongue movements were normal. He was delayed in speech, as well as other milestones such as motor skills and hand use. He was not receiving any regular developmental therapy. He had been tested and found negative for hypothyroidism at birth and again at six months. There had been no cardiac or any other congenital abnormalities detected. The echocardiography had been normal.The ulcer was non-tender, indurated, not bleeding, and was in apposition with two natal teeth in lower central dentition (Figures 1 and 2). There was no regional lymphadenopathy. A clinical diagnosis of Riga-Fede disease due to repetitive trauma resulting from raking movements of the tongue against anterior natal teeth was made. The mother was reassured and the natal teeth were extracted.

## Discussion

The raised edges of Riga-Fede disease resemble a malignant process. Awareness of this benign condition is important as it will avoid unnecessary anxiety and biopsy.

The published literature indicates that at least one fourth of the documented cases of Riga-Fede disease are associated with underlying neuro-developmental disorders. Due to forward and backward movements, the ventral surface of tongue is often the site affected [2]. Encephalopathy, microcephaly, cerebral palsy, familial dysautonomia, Lesch Nyhan syndrome, and Down syndrome are among the neuro-developmental disorders associated with this condition.

Our patient had Down syndrome, which is frequently associated with protrusion and continuous sucking of the tongue, and is well-known to result in a fissured appearance described as ‘scrotal tongue’. This occurs at an older age and was not seen in our patient. Riga-Fede disease caused by underlying neuro-developmental disorders usually develops at older ages and is classified as ‘late onset’ [3].

Another cause of Riga-Fede disease is natal teeth. Natal teeth are not common; the prevalence ranges from 1 in 2000 to 1 in 3000 live births and are commonly lower central incisors [4]. Traumatic ulceration, though rare, is described as ‘early onset’ when seen, and has been reported even as early as the neonatal period. The extraction of natal teeth is recommended due to risk of aspiration or interference with breast feeding.

## Conclusions

Our patient had two causative factors, namely natal teeth and Down syndrome, which is very unusual. The lingual ulcer appeared at around 11 months and we recommend that occurrence of natal teeth alongside another risk factor such as Down syndrome should be considered as an added indicator for the prophylactic extraction of natal teeth.

### Consent

Written informed consent was obtained from the patient’s parents for publication of this case report and the accompanying images. A copy of the written consent is available for review by the Editor-in-Chief of this journal.

## Competing interests

The authors declare that they have no competing interests.

## Authors’ contributions

MPS and IK both provided clinical care to this patient. Both authors were involved in writing the manuscript and in its preparation for publication and contributed intellectually to the article. Both authors read and approved the final manuscript.
